# Di-μ-sulfato-κ^4^ *O*:*O*′-bis­{bis­[3-(2-pyrid­yl)pyrazole]cobalt(II)}

**DOI:** 10.1107/S160053681000797X

**Published:** 2010-03-06

**Authors:** Pei-Xi Lin, Gui-Bin Yang, Zhe An

**Affiliations:** a School of Chemistry and Life Science, Maoming University, Maoming 525000, People’s Republic of China; b School of Chemistry and Life Sciences, Harbin University, Harbin, 150080, People’s Republic of China

## Abstract

In the centrosymmetric binuclear title molecule, [Co_2_(SO_4_)_2_(C_8_H_7_N_3_)_4_], the Co^II^ ion is coordinated by two *N*,*N*′-bidentate 3-(2-pyrid­yl)pyrazole ligands and two sulfate ions, generating a distorted *cis*-CoO_2_N_4_ octa­hedral geometry for the metal atom. The dihedral angles between the pyridine and pyrazole rings in the two ligands are 10.5 (2) and 7.38 (19)°. The bridging sulfate ions generate an eight-membered ring and intra­molecular N—H⋯O hydrogen bonds help to establish the mol­ecular conformation.

## Related literature

For coordination compounds with pyridyl-pyrazolide ligands, see: Ward *et al.* (1998 ▶, 2001 ▶); Zhang *et al.* (2003 ▶).
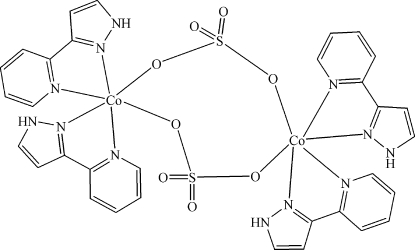


## Experimental

### 

#### Crystal data

[Co_2_(SO_4_)_2_(C_8_H_7_N_3_)_4_]
*M* *_r_* = 890.64
Triclinic, 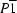

*a* = 8.318 (5) Å
*b* = 9.879 (5) Å
*c* = 11.807 (6) Å
α = 100.342 (8)°
β = 98.820 (9)°
γ = 99.302 (8)°
*V* = 925.2 (9) Å^3^
*Z* = 1
Mo *K*α radiation
μ = 1.08 mm^−1^
*T* = 294 K
0.12 × 0.10 × 0.08 mm

#### Data collection

Bruker APEXII CCD diffractometer
Absorption correction: multi-scan (*SADABS*; Bruker, 2001 ▶) *T*_min_ = 0.882, *T*_max_ = 0.919
4790 measured reflections
3228 independent reflections
2990 reflections with *I* > 2σ(*I*)
*R*_int_ = 0.011

#### Refinement

*R*[*F* ^2^ > 2σ(*F* ^2^)] = 0.041
*wR*(*F* ^2^) = 0.130
*S* = 1.00
3228 reflections
253 parameters
H-atom parameters not refined
Δρ_max_ = 0.61 e Å^−3^
Δρ_min_ = −0.54 e Å^−3^

### 

Data collection: *APEX2* (Bruker, 2004 ▶); cell refinement: *SAINT-Plus* (Bruker, 2001 ▶); data reduction: *SAINT-Plus*; program(s) used to solve structure: *SHELXS97* (Sheldrick, 2008 ▶); program(s) used to refine structure: *SHELXL97* (Sheldrick, 2008 ▶); molecular graphics: *SHELXTL* (Sheldrick, 2008 ▶); software used to prepare material for publication: *SHELXTL*.

## Supplementary Material

Crystal structure: contains datablocks I, global. DOI: 10.1107/S160053681000797X/hb5349sup1.cif

Structure factors: contains datablocks I. DOI: 10.1107/S160053681000797X/hb5349Isup2.hkl

Additional supplementary materials:  crystallographic information; 3D view; checkCIF report

## Figures and Tables

**Table:** 

Co1—O2^i^	2.074 (3)
Co1—O4	2.097 (3)
Co1—N5	2.187 (3)
Co1—N2	2.212 (3)
Co1—N6	2.331 (3)
Co1—N3	2.331 (3)

**Table:** 

N5—Co1—N6	71.51 (10)
N2—Co1—N3	71.12 (10)

Symmetry code: (i) 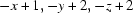.

**Table 2:** Hydrogen-bond geometry (Å, °)

*D*—H⋯*A*	*D*—H	H⋯*A*	*D*⋯*A*	*D*—H⋯*A*
N1—H1*A*⋯O1	0.86	1.98	2.772 (4)	152
N4—H4⋯O1^i^	0.86	1.96	2.761 (4)	155

Symmetry code: (i) 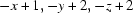.

**Fig. 1.**
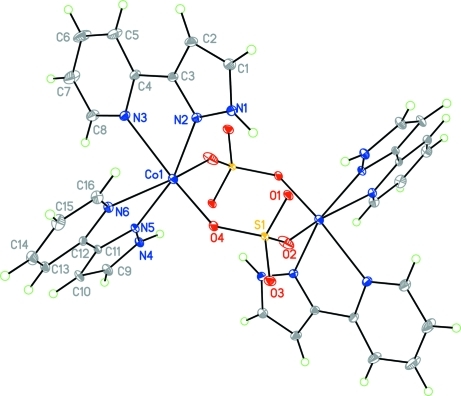
The molecular structure of (I) with displacement ellipsoids drawn at the 30% probability level; H atoms are given as spheres of arbitrary radius. Unlabelled atoms are generated by the symmetry operation (1–x, 2–y, 2–z).

## supplementary crystallographic information

### Comment

The tridentate ligand 3-(2-pyridyl)pyrazole and its derivatives have been used widely in the construction of supramolecular architectures by way of metal-organic coordination (Ward *et al.* 1998; 2001; Zhang *et al.* 2003).

As a continuation of these studies, we now report the crystal structure of the title complex, (I). As shown in Figure 1, two Co(II) cations chelated by two 3-(2-Pyridyl)pyrazole) are linked by two sulfate ions to form one circle in which the cobalt ion is hexacoordinated by two 3-(2-Pyridyl)pyrazole) ligands and two O from two sulfate ions (Table 1).

### Experimental

A mixture of cobalt sulfate heptahydrate (1 mmol, 0.25 g), sodium hydroxide (0.04 g, 1 mmol) and 3-(2-pyridyl)pyrazole (1 mmol, 0.15 g) and water (15 ml) was stirred for 30 min in air. The mixture was then transferred to a 25 ml Teflon-lined hydrothermal bomb. The bomb was kept at 433 K for 72 h under autogenous pressure. Upon cooling, red blocks of (I) were obtained from the reaction mixture.

### Refinement

All hydrogen atoms bound to carbon were refined using a riding model with C—H = 0.93 Å and Uiso(H) = 1.2Ueq(C). The H atoms on nitrogen atoms were refined using a riding model with N—H = 0.86 Å and Uiso(H) = 1.2Ueq(C).

### Crystal data

**Table:**

[Co_2_(SO_4_)_2_(C_8_H_7_N_3_)_4_]	*Z* = 1
*M**_r_* = 890.64	*F*(000) = 454
Triclinic, *P*1	*D*_x_ = 1.599 Mg m^−^^3^
Hall symbol: -P 1	Mo *K*α radiation, λ = 0.71073 Å
*a* = 8.318 (5) Å	Cell parameters from 3228 reflections
*b* = 9.879 (5) Å	θ = 2.1–25.0°
*c* = 11.807 (6) Å	µ = 1.08 mm^−^^1^
α = 100.342 (8)°	*T* = 294 K
β = 98.820 (9)°	Block, red
γ = 99.302 (8)°	0.12 × 0.10 × 0.08 mm
*V* = 925.2 (9) Å^3^	

### Data collection

**Table:**

Bruker APEXII CCD diffractometer	3228 independent reflections
Radiation source: fine-focus sealed tube	2990 reflections with *I* > 2σ(*I*)
graphite	*R*_int_ = 0.011
phi and ω scans	θ_max_ = 25.0°, θ_min_ = 2.1°
Absorption correction: multi-scan (*SADABS*; Bruker, 2001)	*h* = −9→9
*T*_min_ = 0.882, *T*_max_ = 0.919	*k* = −11→11
4790 measured reflections	*l* = −14→10

### Refinement

**Table:**

Refinement on *F*^2^	Primary atom site location: structure-invariant direct methods
Least-squares matrix: full	Secondary atom site location: difference Fourier map
*R*[*F*^2^ > 2σ(*F*^2^)] = 0.041	Hydrogen site location: inferred from neighbouring sites
*wR*(*F*^2^) = 0.130	H-atom parameters not refined
*S* = 1.00	*w* = 1/[σ^2^(*F*_o_^2^) + (0.078*P*)^2^ + 1.7757*P*] where *P* = (*F*_o_^2^ + 2*F*_c_^2^)/3
3228 reflections	(Δ/σ)_max_ = 0.005
253 parameters	Δρ_max_ = 0.61 e Å^−^^3^
0 restraints	Δρ_min_ = −0.54 e Å^−^^3^

### Special details

**Table:**

Geometry. All esds (except the esd in the dihedral angle between two l.s. planes) are estimated using the full covariance matrix. The cell esds are taken into account individually in the estimation of esds in distances, angles and torsion angles; correlations between esds in cell parameters are only used when they are defined by crystal symmetry. An approximate (isotropic) treatment of cell esds is used for estimating esds involving l.s. planes.
Refinement. Refinement of *F*^2^ against ALL reflections. The weighted *R*-factor wR and goodness of fit *S* are based on *F*^2^, conventional *R*-factors *R* are based on *F*, with *F* set to zero for negative *F*^2^. The threshold expression of *F*^2^ > σ(*F*^2^) is used only for calculating *R*-factors(gt) etc. and is not relevant to the choice of reflections for refinement. *R*-factors based on *F*^2^ are statistically about twice as large as those based on *F*, and *R*- factors based on ALL data will be even larger.

### Fractional atomic coordinates and isotropic or equivalent isotropic displacement parameters (Å^2^)

**Table:**

	*x*	*y*	*z*	*U*_iso_*/*U*_eq_	
Co1	0.42221 (5)	0.79964 (4)	0.84355 (4)	0.03040 (18)	
C1	−0.0398 (5)	0.9232 (4)	0.7050 (3)	0.0420 (9)	
H1	−0.1395	0.9531	0.7097	0.050*	
C2	0.0198 (5)	0.8857 (4)	0.6063 (3)	0.0448 (9)	
H2	−0.0302	0.8835	0.5298	0.054*	
C3	0.1714 (4)	0.8511 (4)	0.6431 (3)	0.0318 (7)	
C4	0.2985 (4)	0.8065 (4)	0.5790 (3)	0.0371 (8)	
C5	0.2946 (6)	0.8116 (6)	0.4641 (4)	0.0636 (13)	
H5	0.2092	0.8431	0.4223	0.076*	
C6	0.4197 (7)	0.7690 (8)	0.4113 (4)	0.090 (2)	
H6	0.4207	0.7721	0.3332	0.108*	
C7	0.5414 (7)	0.7226 (7)	0.4750 (4)	0.0846 (19)	
H7	0.6261	0.6922	0.4406	0.102*	
C8	0.5382 (5)	0.7212 (5)	0.5893 (4)	0.0530 (11)	
H8	0.6220	0.6890	0.6322	0.064*	
C9	0.8750 (4)	0.6412 (4)	0.9368 (4)	0.0403 (8)	
H9	0.9883	0.6490	0.9619	0.048*	
C10	0.7598 (4)	0.5198 (3)	0.9034 (3)	0.0368 (8)	
H10	0.7774	0.4287	0.9004	0.044*	
C11	0.6107 (4)	0.5620 (3)	0.8748 (3)	0.0249 (6)	
C12	0.4408 (4)	0.4824 (3)	0.8353 (3)	0.0252 (6)	
C13	0.4011 (4)	0.3396 (3)	0.8316 (3)	0.0342 (7)	
H13	0.4828	0.2898	0.8524	0.041*	
C14	0.2386 (5)	0.2743 (4)	0.7967 (4)	0.0490 (10)	
H14	0.2081	0.1786	0.7933	0.059*	
C15	0.1224 (5)	0.3493 (4)	0.7670 (4)	0.0517 (10)	
H15	0.0114	0.3059	0.7434	0.062*	
C16	0.1705 (4)	0.4906 (4)	0.7721 (3)	0.0425 (8)	
H16	0.0898	0.5414	0.7512	0.051*	
N1	0.0705 (3)	0.9095 (3)	0.7943 (2)	0.0293 (6)	
H1A	0.0596	0.9270	0.8664	0.035*	
N2	0.2007 (3)	0.8649 (3)	0.7574 (2)	0.0283 (6)	
N3	0.4193 (4)	0.7642 (3)	0.6426 (2)	0.0355 (6)	
N4	0.7960 (3)	0.7468 (3)	0.9271 (2)	0.0298 (6)	
H4	0.8437	0.8338	0.9435	0.036*	
N5	0.6333 (3)	0.7005 (3)	0.8887 (2)	0.0249 (5)	
N6	0.3280 (3)	0.5577 (3)	0.8057 (2)	0.0294 (6)	
O1	0.1481 (3)	0.9642 (2)	1.03701 (19)	0.0307 (5)	
O2	0.4260 (3)	1.0067 (3)	1.1403 (3)	0.0592 (9)	
O3	0.2278 (3)	0.8263 (3)	1.1747 (2)	0.0411 (6)	
O4	0.3184 (3)	0.7973 (3)	0.9944 (2)	0.0429 (6)	
S1	0.28057 (8)	0.89939 (7)	1.08803 (6)	0.0206 (2)	

### Atomic displacement parameters (Å^2^)

**Table:**

	*U*^11^	*U*^22^	*U*^33^	*U*^12^	*U*^13^	*U*^23^
Co1	0.0288 (3)	0.0295 (3)	0.0343 (3)	0.00912 (19)	0.00506 (19)	0.00780 (19)
C1	0.0307 (18)	0.055 (2)	0.044 (2)	0.0196 (16)	0.0048 (15)	0.0114 (17)
C2	0.041 (2)	0.066 (3)	0.0311 (18)	0.0229 (18)	0.0006 (15)	0.0126 (17)
C3	0.0290 (17)	0.0389 (18)	0.0276 (16)	0.0088 (14)	0.0025 (13)	0.0076 (13)
C4	0.0332 (18)	0.052 (2)	0.0267 (17)	0.0145 (16)	0.0054 (14)	0.0047 (15)
C5	0.056 (3)	0.114 (4)	0.031 (2)	0.042 (3)	0.0086 (18)	0.018 (2)
C6	0.083 (4)	0.175 (7)	0.033 (2)	0.069 (4)	0.022 (2)	0.028 (3)
C7	0.071 (3)	0.158 (6)	0.045 (3)	0.066 (4)	0.029 (2)	0.019 (3)
C8	0.042 (2)	0.084 (3)	0.040 (2)	0.031 (2)	0.0119 (17)	0.012 (2)
C9	0.0263 (17)	0.0351 (19)	0.060 (2)	0.0111 (14)	0.0031 (16)	0.0110 (17)
C10	0.0328 (18)	0.0241 (16)	0.055 (2)	0.0121 (13)	0.0036 (15)	0.0092 (15)
C11	0.0291 (16)	0.0198 (14)	0.0280 (15)	0.0069 (12)	0.0082 (12)	0.0060 (11)
C12	0.0301 (16)	0.0218 (15)	0.0248 (14)	0.0052 (12)	0.0095 (12)	0.0040 (11)
C13	0.043 (2)	0.0224 (15)	0.0372 (18)	0.0033 (14)	0.0113 (15)	0.0063 (13)
C14	0.057 (3)	0.0294 (18)	0.055 (2)	−0.0076 (17)	0.0132 (19)	0.0058 (16)
C15	0.034 (2)	0.048 (2)	0.061 (3)	−0.0140 (17)	0.0075 (18)	0.0009 (19)
C16	0.0308 (18)	0.043 (2)	0.050 (2)	0.0051 (15)	0.0048 (16)	0.0058 (17)
N1	0.0252 (13)	0.0342 (14)	0.0300 (14)	0.0082 (11)	0.0074 (11)	0.0068 (11)
N2	0.0234 (13)	0.0339 (14)	0.0284 (14)	0.0061 (11)	0.0051 (10)	0.0080 (11)
N3	0.0339 (15)	0.0463 (17)	0.0280 (14)	0.0131 (13)	0.0062 (12)	0.0068 (12)
N4	0.0246 (13)	0.0223 (13)	0.0421 (15)	0.0037 (10)	0.0055 (11)	0.0075 (11)
N5	0.0231 (13)	0.0203 (12)	0.0336 (14)	0.0067 (10)	0.0072 (10)	0.0081 (10)
N6	0.0250 (13)	0.0277 (13)	0.0351 (14)	0.0048 (11)	0.0064 (11)	0.0051 (11)
O1	0.0285 (11)	0.0287 (11)	0.0361 (12)	0.0150 (9)	−0.0003 (9)	0.0068 (9)
O2	0.0386 (15)	0.0315 (13)	0.094 (2)	−0.0101 (11)	−0.0226 (15)	0.0237 (14)
O3	0.0504 (15)	0.0437 (14)	0.0438 (14)	0.0197 (12)	0.0231 (12)	0.0255 (11)
O4	0.0641 (17)	0.0460 (14)	0.0352 (13)	0.0374 (13)	0.0234 (12)	0.0156 (11)
S1	0.0194 (4)	0.0186 (4)	0.0260 (4)	0.0064 (3)	0.0043 (3)	0.0078 (3)

### Geometric parameters (Å, °)

**Table:**

Co1—O2^i^	2.074 (3)	C9—H9	0.9300
Co1—O4	2.097 (3)	C10—C11	1.384 (5)
Co1—N5	2.187 (3)	C10—H10	0.9300
Co1—N2	2.212 (3)	C11—N5	1.327 (4)
Co1—N6	2.331 (3)	C11—C12	1.463 (4)
Co1—N3	2.331 (3)	C12—N6	1.332 (4)
C1—N1	1.329 (4)	C12—C13	1.387 (4)
C1—C2	1.351 (5)	C13—C14	1.366 (5)
C1—H1	0.9300	C13—H13	0.9300
C2—C3	1.386 (5)	C14—C15	1.351 (6)
C2—H2	0.9300	C14—H14	0.9300
C3—N2	1.312 (4)	C15—C16	1.376 (6)
C3—C4	1.469 (5)	C15—H15	0.9300
C4—N3	1.328 (4)	C16—N6	1.334 (4)
C4—C5	1.362 (5)	C16—H16	0.9300
C5—C6	1.376 (6)	N1—N2	1.337 (4)
C5—H5	0.9300	N1—H1A	0.8600
C6—C7	1.357 (7)	N4—N5	1.336 (4)
C6—H6	0.9300	N4—H4	0.8600
C7—C8	1.357 (6)	O1—S1	1.466 (2)
C7—H7	0.9300	O2—S1	1.446 (3)
C8—N3	1.336 (5)	O2—Co1^i^	2.074 (3)
C8—H8	0.9300	O3—S1	1.436 (2)
C9—N4	1.332 (4)	O4—S1	1.466 (2)
C9—C10	1.363 (5)		
			
O2^i^—Co1—O4	109.51 (13)	N5—C11—C10	110.9 (3)
O2^i^—Co1—N5	92.56 (11)	N5—C11—C12	117.4 (3)
O4—Co1—N5	99.64 (10)	C10—C11—C12	131.7 (3)
O2^i^—Co1—N2	93.38 (11)	N6—C12—C13	122.9 (3)
O4—Co1—N2	89.94 (10)	N6—C12—C11	115.1 (3)
N5—Co1—N2	166.34 (10)	C13—C12—C11	122.0 (3)
O2^i^—Co1—N6	161.04 (12)	C14—C13—C12	118.1 (3)
O4—Co1—N6	83.92 (10)	C14—C13—H13	121.0
N5—Co1—N6	71.51 (10)	C12—C13—H13	121.0
N2—Co1—N6	100.13 (10)	C15—C14—C13	119.7 (3)
O2^i^—Co1—N3	87.49 (13)	C15—C14—H14	120.1
O4—Co1—N3	155.57 (11)	C13—C14—H14	120.1
N5—Co1—N3	96.88 (10)	C14—C15—C16	119.2 (4)
N2—Co1—N3	71.12 (10)	C14—C15—H15	120.4
N6—Co1—N3	84.40 (10)	C16—C15—H15	120.4
N1—C1—C2	107.2 (3)	N6—C16—C15	122.7 (4)
N1—C1—H1	126.4	N6—C16—H16	118.7
C2—C1—H1	126.4	C15—C16—H16	118.7
C1—C2—C3	105.4 (3)	C1—N1—N2	111.2 (3)
C1—C2—H2	127.3	C1—N1—H1A	124.4
C3—C2—H2	127.3	N2—N1—H1A	124.4
N2—C3—C2	110.2 (3)	C3—N2—N1	106.0 (3)
N2—C3—C4	117.7 (3)	C3—N2—Co1	119.5 (2)
C2—C3—C4	132.1 (3)	N1—N2—Co1	134.3 (2)
N3—C4—C5	122.7 (3)	C4—N3—C8	117.8 (3)
N3—C4—C3	114.8 (3)	C4—N3—Co1	116.2 (2)
C5—C4—C3	122.5 (3)	C8—N3—Co1	125.5 (2)
C4—C5—C6	118.6 (4)	C9—N4—N5	111.4 (3)
C4—C5—H5	120.7	C9—N4—H4	124.3
C6—C5—H5	120.7	N5—N4—H4	124.3
C7—C6—C5	119.0 (4)	C11—N5—N4	105.3 (2)
C7—C6—H6	120.5	C11—N5—Co1	119.8 (2)
C5—C6—H6	120.5	N4—N5—Co1	134.78 (19)
C8—C7—C6	119.3 (4)	C16—N6—C12	117.4 (3)
C8—C7—H7	120.3	C16—N6—Co1	126.2 (2)
C6—C7—H7	120.3	C12—N6—Co1	115.8 (2)
N3—C8—C7	122.5 (4)	S1—O2—Co1^i^	153.33 (18)
N3—C8—H8	118.7	S1—O4—Co1	137.65 (16)
C7—C8—H8	118.7	O3—S1—O2	110.08 (18)
N4—C9—C10	107.6 (3)	O3—S1—O4	108.22 (15)
N4—C9—H9	126.2	O2—S1—O4	110.2 (2)
C10—C9—H9	126.2	O3—S1—O1	110.61 (15)
C9—C10—C11	104.7 (3)	O2—S1—O1	109.36 (15)
C9—C10—H10	127.7	O4—S1—O1	108.40 (14)
C11—C10—H10	127.7		

Symmetry codes: (i) −*x*+1, −*y*+2, −*z*+2.

### Hydrogen-bond geometry (Å, °)

**Table:**

*D*—H···*A*	*D*—H	H···*A*	*D*···*A*	*D*—H···*A*
N1—H1A···O1	0.86	1.98	2.772 (4)	152
N4—H4···O1^i^	0.86	1.96	2.761 (4)	155

Symmetry codes: (i) −*x*+1, −*y*+2, −*z*+2.
